# Supplementary material for: Comparison between Peritumoral and Intratumoral Budding in Colorectal Cancer
Source: Biomedicines. 2024 Jan 17;12(1):212. doi: 10.3390/biomedicines12010212 (PMC10813595; doi:10.3390/biomedicines12010212)
Supplement: Supplementary file 1 [file biomedicines-12-00212-s001.zip › biomedicines-2784353-supplementary.pptx]

## Slide 1
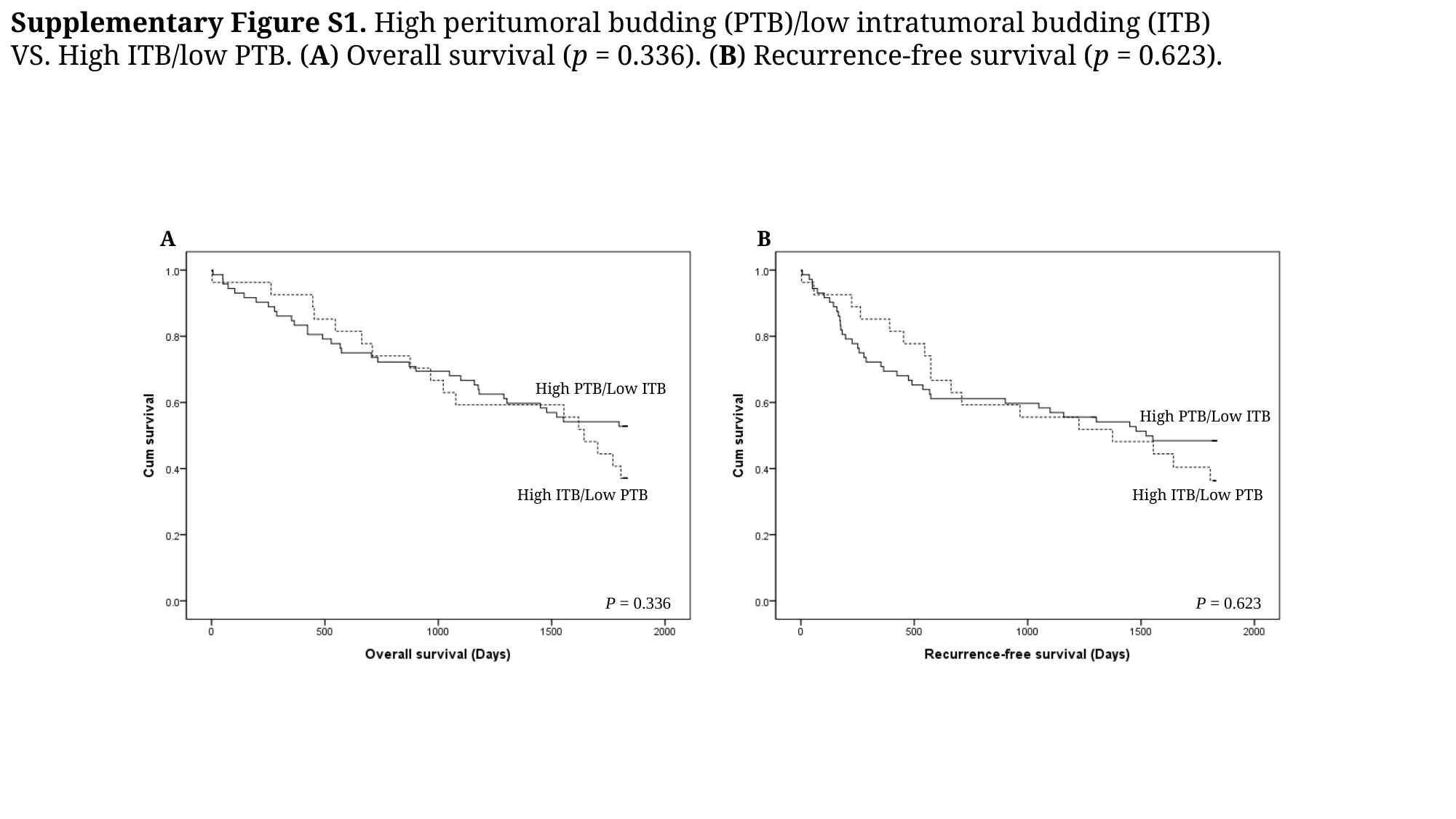

Supplementary Figure S1. High peritumoral budding (PTB)/low intratumoral budding (ITB) VS. High ITB/low PTB. (A) Overall survival (p = 0.336). (B) Recurrence-free survival (p = 0.623).
A
B
High PTB/Low ITB
High PTB/Low ITB
High ITB/Low PTB
High ITB/Low PTB
P = 0.336
P = 0.623
